# Successful curative resection of gallbladder cancer following S-1 chemotherapy: A case report and review of the literature

**DOI:** 10.3892/ol.2014.2565

**Published:** 2014-09-26

**Authors:** TAKAHIRO EINAMA, KOICHIRO UCHIDA, MASAHIKO TANIGUCHI, YU OTA, KENJI WATANABE, KOJI IMAI, HIDENORI KARASAKI, ATSUSHI CHIBA, KENSUKE OIKAWA, NAOYUKI MIYOKAWA, HIROYUKI FURUKAWA

**Affiliations:** 1 Division of Gastroenterological and General Surgery, Asahikawa Medical University, Asahikawa, Hokkaido 078-8510, Japan; 2 Department of Surgery, Hokkaido Social Work Association Obihiro Hospital, Obihiro, Hokkaido 080-0805, Japan; 3 Digestive Disease Center, Asahikawa City Hospital, Hokkaido 070-8610, Japan; 4 Department of Surgical Pathology, Asahikawa Medical University, Asahikawa, Hokkaido 078-8510, Japan

**Keywords:** gallbladder cancer, S-1, gene expression

## Abstract

The symptoms of gallbladder cancer (GBC) are vague and non-specific. Therefore, GBC is often detected at an advanced or metastatic stage. The most effective treatment for GBC is surgical resection, however the majority of GBC cases are unresectable at the time of diagnosis. Therefore, numerous GBC patients undergo chemotherapy. This study reports the case of a 60-year-old female with GBC who underwent successful surgical curative resection following a single dose of the chemotherapeutic agent, S-1, twice daily for 4 weeks followed by a 14-day rest period for 36 months. S-1 is a novel orally administered drug composed of a combination of the 5-fluorouracil (5-FU) prodrug, tegafur, 5-chloro-2,4-dihydroxypyridine (CDHP) and oteracil potassium in a 1:0.4:1 molar concentration ratio. The focus of the present study was the candidate factors that affect the therapeutic efficacy of S-1-based chemotherapy. In particular, the gene expression involved in the S-1 metabolic pathway was investigated by assessing the intratumoral dihydropyrimidine dehydrogenase (*DPD*)*,* thymidylate synthase (*TS*) and orotate phosphoribosyltransferase gene expression. The surgical specimen exhibited high intratumoral *DPD* gene expression levels compared with those observed in previously reported non S-1 responsive cases of biliary tract cancer. Due to the results obtained in the current study, we hypothesize that CDHP enhanced the antitumor efficacy of 5-FU by inhibiting the excess DPD protein produced by the tumor.

## Introduction

Gallbladder cancer (GBC) is the most aggressive type of biliary tract cancer (BTC) and exhibits the shortest median survival time worldwide. Complete surgical resection offers the only chance of complete remission; however, GBC is characterized by local invasion, extensive regional lymph node metastasis, vascular encasement and distant metastases. Therefore, only 10% of patients present with early-stage disease are considered to be candidates for surgery (1). Therefore, chemotherapy serves as the primary treatment in the majority of GBC cases. Previous studies have found that patients with metastatic GBC who receive palliative therapy have a median survival time of approximately six months (2,3). Therefore, more effective chemotherapies are required for the management of GBC.

S-1 is a novel orally administered drug composed of a combination of the 5-fluorouracil (5-FU) prodrug, tegafur (FT), 5-chloro-2,4-dihydroxypyridine (CDHP) and oteracil potassium (OXO) in a 1:0.4:1 molar concentration ratio (4). Based on the results obtained from randomized phase III trials, S-1 has become a key drug in the treatment of advanced gastric cancer in Japan and is considered to be the standard option for chemotherapy (5,6). Furthermore, gemcitabine and S-1 have also been approved for clinical use in the treatment of BTC by the Ministry of Health, Labour and Welfare in August 2007 (7).

The current study presents a case of GBC in which the patient underwent successful surgical curative resection following a single dose of S-1. The focus of this study was the candidate characteristics that affect the therapeutic efficacy of S-1-based chemotherapy. In particular, the gene expression involved in the S-1 metabolic pathway was investigated by analyzing dihydropyrimidine dehydrogenase (*DPD*), thymidylate synthase (*TS*) and orotate phosphoribosyltransferase (*OPRT*) gene expression. The patient provided full written informed consent prior to the initiation of the study.

## Case report

**Figure 1:**
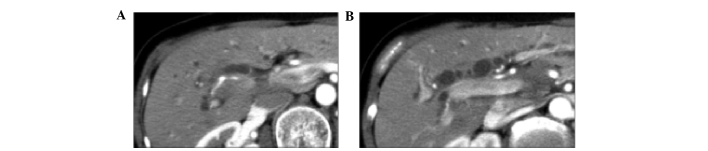
Computed tomography scans revealing (A) direct invasion of the right hepatic artery and (B) perineural invasion of the common hepatic and celiac arteries.

A 60-year-old female was admitted to Asahikawa City Hospital (Asahikawa, Japan) with a primary complaint of jaundice in 2008. The patient’s medical history was unremarkable and, with the exception of the jaundice, the physical examination revealed no abnormalities. The results from the laboratory tests indicated abnormal values for total bilirubin (7.9 mg/dl; normal range, 0.2–1.0 mg/dl), serum glutamic oxaloacetic transaminase (124 IU/l; normal range, 6–40 IU/l) and serum glutamic pyruvic transaminase (228 IU/l; normal range, 6–37 IU/l). On admission, tumor marker levels were as follows: Carcinoembryonic antigen, 21.1 ng/ml (upper normal limit, 4.9 ng/ml) and carbohydrate antigen 19–9, 107.6 IU/ml (upper normal limit, 39 IU/ml). An abdominal computed tomography (CT) scan revealed the presence of advanced GBC, which had invaded the liver, as well as regional lymph node metastasis and perineural invasion of the common hepatic and celiac arteries (Fig. 1). The tumor was considered to be inoperable due to the presence of perineural invasion of the common hepatic and celiac arteries. With informed consent from the patient, chemotherapy with S-1 was initiated at a dose of 100 mg twice daily for four weeks, followed by a 14-day rest period, for a total of 25 cycles.

### Clinical course of treatment

**Figure 2:**
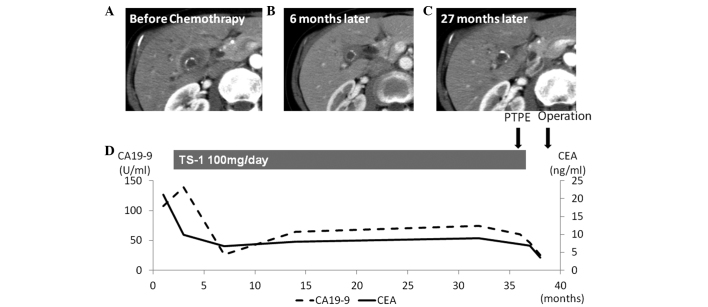
Changes in the patient CEA and CA19-9 levels (A) prior to chemotherapy and (B)six and (C) 27 months following chemotherapy, and computed tomography findings. (D) Clinical course of S-1 treatment. CEA, carcinoembryonic antigen; CA19-9, carbohydrate antigen 19-9; TS-1, thymidylate synthase; PTPE, percutaneous transhepatic portal embolization.

**Figure 3:**
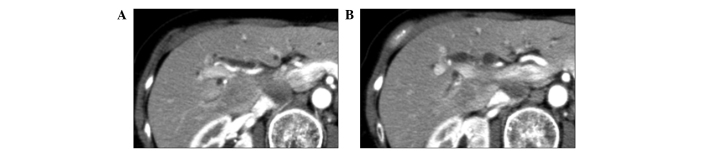
Changes after chemotherapy (A) in the gallbladder tumor and (B) in terms of neural invasion, shown by computed tomography imaging. (A) A clear reduction in size and decreased invasion was observed in the right hepatic artery. (B) Decreased perineural invasion of the common hepatic and celiac arteries was observed.

The patient was followed up every six weeks for a total of 36 months. The tumor marker levels decreased after two months(Fig. 2), and a CT scan demonstrated a clear reduction in size in the regions of the liver that were invaded by the GBC, as well as those affected by lymph node metastasis and perineural invasion (Fig. 3). A partial response was maintained for over 30 months and, subsequently, surgical resection with curative intent was planned for 36 months following the initiation of treatment with S-1.

**Figure 4:**
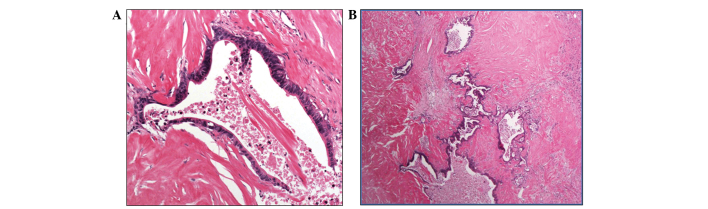
Pathological examination demonstrated that the tumor was (A) a scirrhous adenocarcinoma and (B) predominantly consisted of fibrosis and necrosis tissue [stain, hematoxylin and eosin; magnification (A) ×200 and (B) ×40.

The patient underwent percutaneous transhepatic portal embolization and, three weeks following this, a right lobectomy with extrabiliary duct resection and lymphadenectomy was performed. The pathological findings of the tumor were compatible with a diagnosis of adenocarcinoma of the gallbladder. The tumor had directly invaded the liver and cancer cells were found in the perineural area; however there was no metastatic lesion in the liver and no regional lymph node metastasis. All surgical margins were negative (Fig. 4) and a pathological R0 resection was achieved. The postoperative course was uneventful, and the patient was discharged 25 days following surgery.

### Intratumoral gene expression levels of DPD, OPRT and TS

The resected gallbladder specimen was analyzed to determine the intratumoral gene expression levels of DPD, OPRT and TS, which encode the corresponding key enzymes that are involved in the metabolism of 5-FU (8,9). The expression levels of these genes were all measured by Response Genetics (Los Angels, CA, USA) using the Danenburg Tumor Profile method and laser capture microdissection, as described previously (10,11). The specimen exhibited high intratumoral *DPD* gene expression levels compared with those observed in the BTC cases who were non-responders of S-1 treatment (Table I).

## Discussion

The symptoms of GBC are vague and non-specific; therefore, GBC is often detected at an advanced or metastatic stage. The most effective treatment for GBC is surgical resection; however, the majority of GBCs are unresectable at the time of diagnosis (1,12,13) and, hence, numerous GBC patients undergo chemotherapy (14).

In colorectal cancer, primary systemic chemotherapy appears to be a promising approach to the management of patients with initially unresectable liver metastases, as it leads to a reduction in the lesion size, which facilitates the surgical resection in a high proportion of cases (15). Patients who are able to undergo complete resection following chemotherapy tend to achieve improved outcomes (15–17).

A review of the literature identified only three case reports with regard to surgical resection following chemotherapy for unresectable GBC (Table II) (14,18,19). Two of the four cases survived for >1 year without recurrence (one survived for a year and no information is available for one case). Compared with unresectable GBC cases, the patients reported in these cases had a good prognosis. In the present case, surgery was performed three years following treatment with S-1. To date, no evidence of recurrence has been observed for 30 months following surgery.

The novel antitumor drug S-1 contains a prodrug of 5-FU and was developed based on the biochemical effects of CDHP, a DPD inhibitor, and OXO, an OPRT inhibitor, in the small intestine. The principal roles of these modulators are to inhibit the degradation of 5-FU and to protect against 5-FU-induced gastrointestinal toxicity. TS is a major target of 5-FU, which inhibits DNA synthesis. High TS activity in cancer tissue is considered to reduce the efficacy of 5-FU and it is likely that the DPD mRNA level is also a significant predictor of the response to 5-FU. Low TS and DPD expression levels are associated with poor outcomes in colorectal cancer patients who are treated with surgery alone, whereas these low expression levels are associated with improved outcomes in patients who are treated with 5-FU chemotherapy (20).

The tumor tissue of the patient in the present case and of a patient in a previous case (14) exhibited markedly higher DPD mRNA levels compared with those observed in two BTC patients who did not respond to S-1 treatment (Table I). Conversely, the use of a 5-FU agent in the absence of CDHP is likely to exhibit a decrease in the efficacy, with the agent rapidly becoming inactive due to degradation by the excess DPD produced by the tumor. In the current study, while no conclusive evidence was obtained that the S-1 treatment resulted in the downstaging of the cancer, it is reasonable to speculate that the CDHP in S-1 was significant, and may have enhanced the antitumor efficacy of 5-FU through the inhibition of the excess DPD produced by the tumor, although, additional studies may be required to confirm this.

In conclusion, the current study reports the case of a patient with advanced GBC for whom the anticancer agent, S-1, was effective and a pathological R0 resection was achieved. The results of the present study indicate that the CDHP in S-1 may enhance the antitumor effect of 5-FU by inhibiting the excess DPD protein produced by the tumor. The use of S-1 in patients with GBC warrants further clinical studies. The present study suggests that the CDHP in S-1 may have enhanced the antitumor efficacy of 5-FU by inhibiting the excess DPD produced by the tumor. Analysis of the intratumoral gene expression levels of DPD may be useful to predict the efficacy of S-1 treatment.

## Figures and Tables

**Table I:** Intratumoral *DPD, TS* and *OPRT* gene expression in biliary tract cancer patients.

Author (ref.)	TS	DPD	OPRT
Present case	3.26	8.21	0.91
Kitajima *et al* (14)	1.88	8.21	0.91
Case 1	14.42	3.16	1.35
Case 2	2.73	2.78	0.73

TS, thymidylate synthase; DPD, dihydropyrimidine dehydrogenase; OPRT, orotate phosphoribosyltransferase. Case 1, 39 year-old female: intrahepatic bile duct cancer, post right lobectomy with liver and bone metastasis; case 2, 71 year-old-male: extrahepatic bile duct cancer, post pylorus preserving pancreatoduodenectomy with a recurrence of supra mesenteric artery nerve plexus. Case 1 and case 2 patients received treatment with S-1 and had progressive disease.

**Table II:** Surgical resection following chemotherapy for unresectable gallbladder cancer.

Author (ref.)	Regime	Time to surgery after chemotherapy, months	Regime after surgery	Prognosis
Kitajima *et al* (14)	S-1	8	Unknown	Unknown
Morimoto *et al* (18)	Gem	12	Gem	No recurrence for 20 months
Takita *et al* (19)	Gem + S-1	9	S-1	No recurrence for 12 months
Present case	S-1	36	S-1	No recurrence for 30 months

Gem, gemcitabine.
